# A rare manifestation of STING-associated vasculopathy with onset in infancy: a case report

**DOI:** 10.1186/s12969-023-00934-4

**Published:** 2024-01-04

**Authors:** Sophia Weidler, Sarah Koss, Christine Wolf, Nadja Lucas, Jürgen Brunner, Min Ae Lee-Kirsch

**Affiliations:** 1 Department of Pediatrics, Medizinische Fakultät Carl Gustav Carus, Technische Universität Dresden, Dresden, Germany; 2 Department of Pediatrics, Innsbruck Medical University, Innsbruck, Austria; 3 Faculty of Medicine and Dentistry, Danube Private University, 3500 Krems, Austria

**Keywords:** STING-associated vasculopathy with onset in infancy, Type I interferon, Stimulator of interferon genes, Interstitial lung disease, Alopecia, Chilblain lupus, Autoinflammation, Autoimmunity

## Abstract

**Background:**

STING-associated vasculopathy with onset in infancy (SAVI) is a rare type I interferonopathy caused by heterozygous variants in the *STING* gene. In SAVI, *STING* variants confer a gain-of-function which causes overactivation of type I interferon (IFN) signaling leading to autoinflammation and various degrees of immunodeficiency and autoimmunity.

**Case presentation:**

We report the case of a 5 year old child and his mother, both of whom presented with systemic inflammatory symptoms yet widely varying organ involvement, disease course and therapeutic response. Genetic testing revealed a heterozygous STING variant, R281Q, in the child and his mother that had previously been associated with SAVI. However, in contrast to previously reported SAVI cases due to the R281Q variant, our patients showed an atypical course of disease with alopecia totalis in the child and a complete lack of lung involvement in the mother.

**Conclusions:**

Our findings demonstrate the phenotypic breadth of clinical SAVI manifestations. Given the therapeutic benefit of treatment with JAK inhibitors, early genetic testing for SAVI should be considered in patients with unclear systemic inflammation involving cutaneous, pulmonary, or musculoskeletal symptoms, and signs of immunodeficiency and autoimmunity.

## Background

The rare autoinflammatory syndrome STING-associated vasculopathy with onset in infancy (SAVI) was initially reported as novel type I interferonopathy in 2014 [1]. Patients typically present with infancy-onset systemic inflammation with fever episodes and necrotizing cutaneous vasculitis causing extensive tissue damage. However, the main cause of morbidity and mortality and therefore prognosis-determining manifestation is interstitial lung disease leading to end-stage respiratory failure [1]. SAVI is caused by mostly heterozygous de novo gain-of-function variants in the *STING1* gene encoding stimulator of interferon genes. STING functions as the key signaling adapter molecule of the cGAS-STING DNA-sensing pathway which plays an important role in the innate immune defense against viral infections [2]. Upon DNA ligand recognition, cGAS induces formation of the second messenger cGAMP which binds to STING, thereby triggering TBK1-dependent phosphorylation of the transcription factor IRF3. Upon nuclear translocation, activated IRF3 then induces the expression of the IFNα/β genes as well as of numerous IFN-stimulated genes.

**Fig. 1:**
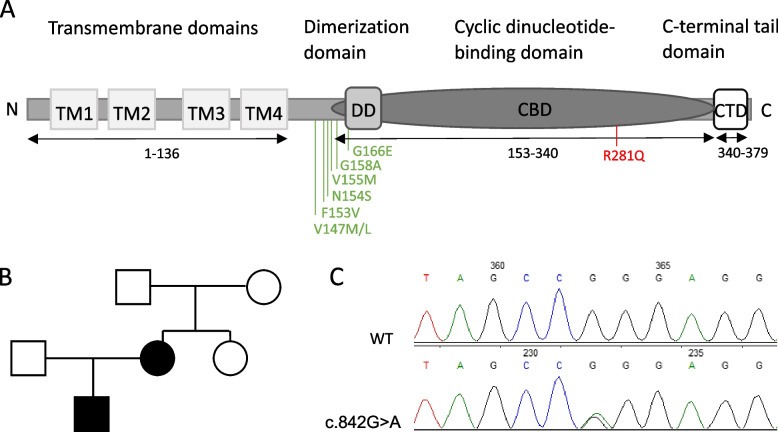
STING protein, pedigree chart and electropherograms. **A**. Schematic illustration of STING protein domains depicting the initially reported and most frequent SAVI disease-causing variants and the R281Q variant described here. **B**. Pedigree chart depicting the index patient (P1) and his mother (P2). **C**. Electropherograms showing the heterozygous *STING1* variant c.842G > A in the lower lane and wildtype in the upper lane

A common feature of SAVI is abnormally enhanced chronic type IFN signaling resulting from STING activation in the absence of cGAMP [1], leading to inadequate inflammatory processes and impairment of immune tolerance. With currently more than 70 reported SAVI cases, the clinical and mutational spectrum has expanded [3]. The first reported cases all involved substitutions of amino acid residues 147, 154, 155 or 166 within the interface of the STING dimer, which alter the linker region between the N-terminal transmembrane domain of STING and the cyclic dinucleotide-binding domain (CBD) or the N-terminal site of the dimerization region of the CBD (Fig. 1A). Only later, genetic variants further downstream involving amino acid residue 281, among others, were reported, delineating a novel region within the CBD as functionally important [4]. Here, we describe a familial case of SAVI due to a heterozygous R281Q STING variant with atypical clinical manifestations including alopecia totalis, chilblain lupus and absence of lung disease.

## Case presentation

### Case 1

**Fig. 2:**
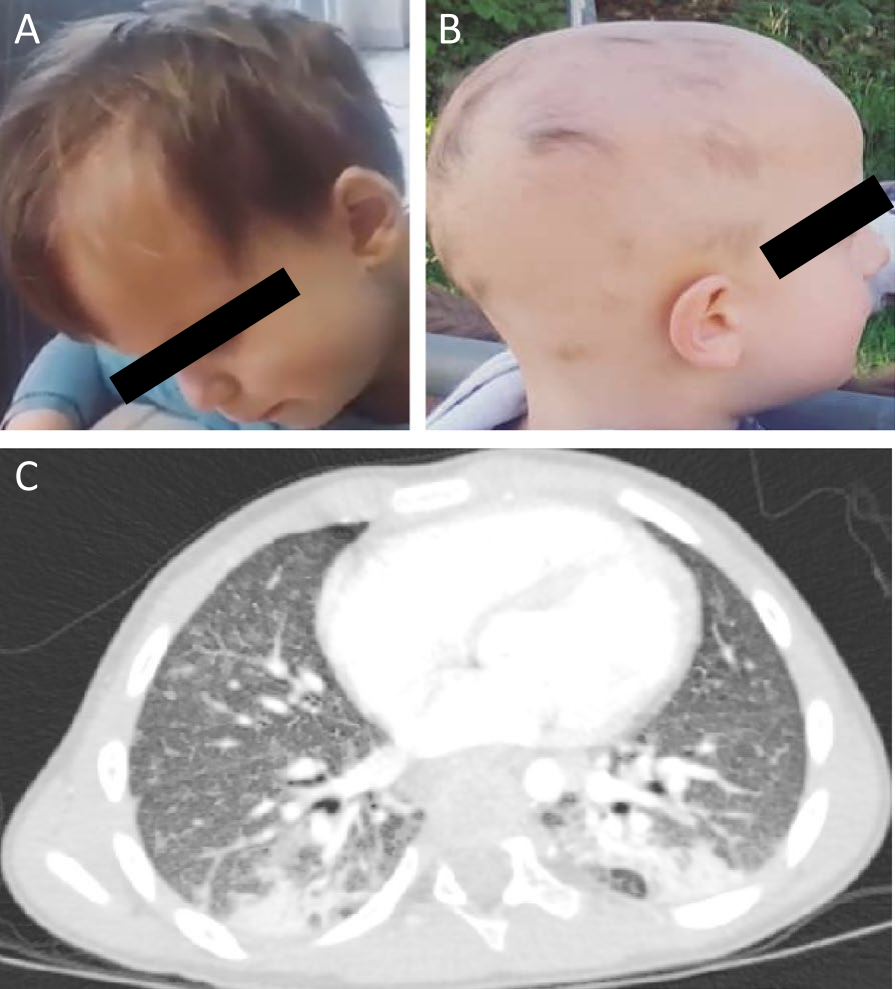
Clinical findings in index patient P1. **A**. Alopecia areata starting from the left forehead. **B**. Alopecia totalis with only isolated hair tufts. **C**. Computed tomography of the chest showing interstitial vasculitic lung disease with ground glass opacities and caudal small nodular parenchymal lesions. Basal bronchopneumonic infiltrates are present on both sides

We report on a male patient (P1) who was born at term to non-consanguineous parents of Austrian origin (Fig. 1B). While birth height and head circumference were within normal limits, the birth weight was at the second percentile. In the first months, weight gain, growth and early psychomotor development were normal. At 6 months, the patient developed eczema and was hospitalized after a third degree anaphylactic reaction after exposure to whey as bath additive. Between 16 to 24 months of age, the patient experienced five febrile seizures in the context of infections. At that point, no further cerebral imaging or cerebrospinal fluid analysis were conducted, because of the uncomplicated and self-limiting nature of the fever seizures with unremarkable findings on neurological examination. Subsequently, respiratory infections recurred on a monthly base. At 3 years of age, the patient was hospitalized three times because of pneumonia with transient hypoxia requiring oxygen therapy and intravenous antibiotics. In addition, due to a protracted course of severe bronchitis, he was treated with inhaled glucocorticoid and β2-sympathomimetic therapy over several months. Also, at 3 years of age, the patient developed alopecia areata (Fig. 2A). At 3.5 years of age, he was admitted to the hospital because of recurring episodes of abdominal pain with loose stools and failure to thrive. Laboratory testing revealed positive fecal occult blood tests and microcytic anemia with a minimal hemoglobin level of 3.3 mmol/L, requiring erythrocyte transfusion and iron substitution. Tissue transglutaminase IgA, anti-gliadin IgA and IgG as well as endomysial antibodies were negative. Stool tests showed normal calprotectin levels but were positive for enterotoxic E. Coli and adenovirus, so that an oral antibiotic course with clarithromycin was started with clinical improvement. In addition, the patient tested positive for antinuclear antibodies (ANA 1:160; normal range < 1:80), perinuclear anti-neutrophil cytoplasmatic antibodies (p-ANCA 1:1280; normal range < 1:40) and myeloperoxidase (MPO) ANCA with > 600 IU/mL (normal range 0.0 – 5.0). Capillary microscopy revealed isolated atypical capillaries of the right third digit. Immunological investigations revealed a slightly increased CD4/CD8 ratio (3.8; reference 0.9–2.6) and mildly reduced levels of complement C3/C3c (108 mg/dl; reference 116.0–210.0), while T cell activation and levels of IgG, IgA and IgM were unremarkable. Further diagnostic work-up for chronic lung disease revealed a normal sweat test with a chloride concentration of < 20 mmol/l. A low-dose chest computed tomography (CT) showed bilateral ground glass opacities with pronounced fine reticular and small nodular vascular lesions compatible with interstitial lung disease (Fig. 2C). On echocardiogram, a hypertrophic right atrium and ventricle with a dilated pulmonary truncus, consistent with pulmonary arterial hypertension (PAH), was noted without the need for therapy. At 4 9/12 years, despite long-term oral iron substitution, microcytic hypochromic anemia persisted requiring an intravenous iron infusion. At that time, the alopecia areata had progressed to alopecia totalis (Fig. 2A, B).

Molecular genetic testing revealed a heterozygous variant in *STING1* (c.842G > A; p.Arg281Gln, R281Q) (Fig. 1C) that had previously been associated with SAVI [4–7]. With the confirmed diagnosis of SAVI at 5 3/12 years of age, the decision was made to treat the patient with the JAK inhibitor tofacitinib. Following oral administration of 0.06 mg/kg tofacitinib every other day over a period of six months, the patient experienced significant clinical improvement with hair growth, weight gain, amelioration of anemia and reduced frequency of infections and pulmonary exacerbations.

### Case 2

**Fig. 3:**
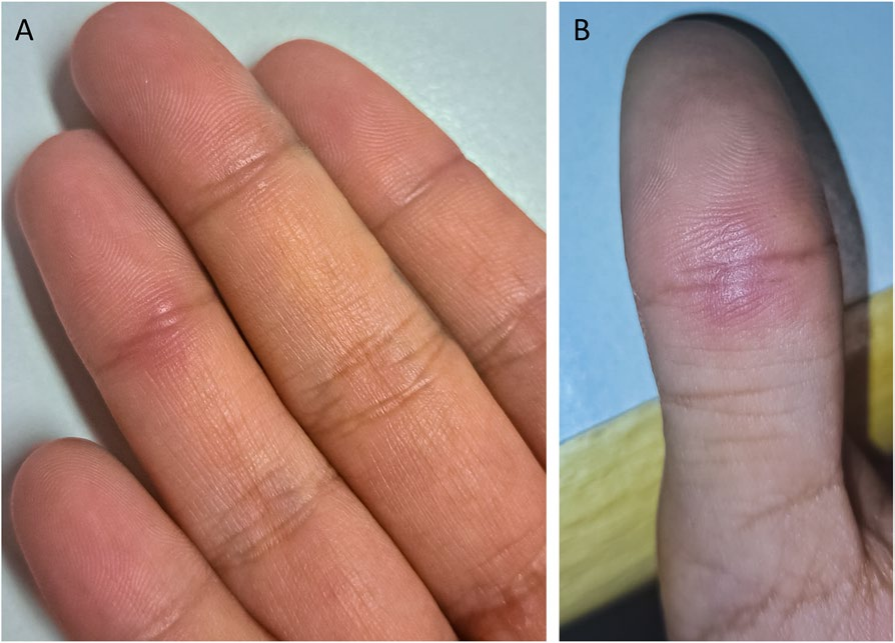
Clinical findings in patient P2. **A**. Erythematous chilblain-like nodules on the palmar site of the fourth digit. **B**. Chilblain lesion on the left thumb

At 17 years of age, the mother of our index patient (P2) presented with reddish skin lesions on the dorsal side of her hands and feet which became increasingly painful nodules (Fig. 3A, B). A skin biopsy showed sparse dermal inflammatory infiltration resembling systemic lupus erythematosus (SLE) or chilblain lupus. At 19 years of age, she started developing arthralgias of her hand and feet about three months after delivery with stiffness and swelling in her fingers and toes, especially the proximal interphalangeal joints. Her joint pain increased significantly limiting her daily activities. Imaging revealed erosive changes in the carpal area but no synovitis or tendosynovitis. Laboratory workup showed an increased erythrocyte sedimentation, while C-reactive protein, immunoglobulins and hemoglobin were within normal limits. p-ANCA were weakly positive (1:40), MPO and proteinase 3 (PR3) ANCA as well as ANA were negative. With the diagnosis of an undifferentiated connective tissue disease and chilblain lupus, immunosuppressive therapy was initiated. Her symptoms improved on prednisone (max. 15 mg/day) but worsened again when tapering was attempted which led to the decision to start treatment with hydroxychloroquine (HCQ 300 mg/day). However, despite HCQ treatment, she still required steroids to control recurrent flares. Immunosuppressive therapy was subsequently switched to methotrexate (MTX) and steroid therapy could briefly be ended, although low inflammatory activity continued. After her son was diagnosed with SAVI, genetic testing of the mother revealed the same heterozygous *STING1* variant, c.842G > A (R281Q) (Fig. 1C). Given incomplete remission under MTX, her therapy was switched to the JAK inhibitor tofacitinib with initially 5 mg/day (0.085 mg/kg) which was not sufficient to control her symptoms. As a the dose increase to 2 × 5 mg/day (0.17 mg/kg) was also not effective, further treatment was terminated on the patient’s side.

Screening for lung involvement, including a chest CT and pulmonary function tests performed at the age of 24 years, did not reveal any sign of interstitial lung disease. Likewise, the patient also showed no clinical signs of pulmonary disease at any time.

## Discussion and conclusions

SAVI is a complex immunodysregulatory disease characterized type I IFN-dependent systemic autoinflammation accompanied by variable degrees of immunodeficiency and autoimmunity. Pulmonary involvement can result in progressive organ injury and high mortality, underlining the importance of early diagnosis and treatment. The high phenotypic variability and clinical overlap with other autoinflammatory syndromes pose a diagnostic challenge. Here we describe two patients from the same family carrying the heterozygous R281Q STING variant who presented with atypical clinical manifestations. To date, 8 patients from 4 families have been described with the heterozygous R281Q STING genotype (Table 1). While inflammatory skin lesions are commonly observed in SAVI patients, our index patient did not show typical SAVI-associated skin lesions, but interstitial lung disease with recurrent respiratory infections. Instead, he presented with alopecia areata that later progressed to alopecia totalis, a feature that has not yet been described in association with the R281Q disease-causing variant. Indeed, the occurrence of alopecia, nailfold capillary changes and low level autoantibodies in the child illustrate the overlap of autoinflammatory and autoimmune features in SAVI. Interestingly, alopecia areata was recently described in a patient with ILD and a biallelic STING variant affecting the same amino acid residue, R281W, which improved under JAK inhibition with ruxolitinib [8]. In contrast, the mother of our index patient, presented with inflammatory chilblain lesions, painful arthritis, severe fatigue but without any lung involvement. Of the previously reported 8 SAVI patients carrying the heterozygous R281Q STING variant, only 4 patients showed skin manifestations including malar rash and teleangiectasia, while 3 presented with joint involvement. Notably, in all of these cases, ILD was the salient clinical manifestation.

**Table 1:** Clinical characteristics of patients with heterozygous p.R281Q disease-causing variant in STING reported in the literature

Reference	Age (age at onset)	Sex	Clinical findings	Laboratory findings	Therapy
Melki et al. [4]	7 y (3 m)	F	severe ILD, PAH, pneumonia teleangiectatic skin lesions on cheeks and nose, recurrent skin infections with poor healing necrotizing granulomatous hepatitis feeding difficulties and growth retardation	inflammatory markers + ANA – ANCA -	limited response to steroids, MTX and TNFα-inhibitor, clinical improvement with ruxolitinib
Li et al. [9]	index: 9 y (shortly after birth)	M	ILD, exertional cough and dyspnea, migratory polyarthralgia of fingers, toes, wrists and knees	RF + ANA + p-ANCA +	no long-term therapy
	brother: 4y (4 y)	M	interstitial changes on chest CT	n/a	none
	father: 33 y (18 y)	M	ILD, exertional dyspnea	n/a	died of respiratory failure at 36 y
Nishida et al. [6]	18y (2 y)	M	juvenile idiopathic arthritis dyspnea, interstitial pneumonia and emphysematous changes, growth retardation	inflammatory markers + ANA + p-ANCA + c-ANCA +	steroids, MTX, azathioprine, TNFα-inhibitor with good response of joint symptoms but progression of lung disease
Wang et al. [7]	index: 37 y (19 y)	M	advanced ILD, pneumonia, PAH, clubbing, nail dystrophy	inflammatory markers + ANA + multiple auto-antibodies +	antibiotics and steroids without improvement; died at 37 y; 4 m after ruxolitinib was started
	son: 13 y (2 y)	M	ILD, migratory polyarthritis, malar rash, growth retardation	inflammatory markers + ANA +	ruxolitinib without improvement
	son: 6y (2 y)	M	ILD, teleangiectasias	inflammatory markers + ANA +	none

*y* years, *m* months, *M* male, *F* female, *ILD* interstitial lung disease, *PAH* pulmonary artery hypertension, *RF* rheumatoid factor, *ANA* antinuclear antibodies, *n/a* not available, *p-ANCA* perinuclear anti-neutrophil cytoplasmic antibodies, *TNFα* tumor necrosis factor-α, *c-ANCA* anti-neutrophil cytoplasmic antibodies, *MTX* methotrexate, + positive test,—negative test

While the exact mechanisms of lung injury in SAVI have not yet been fully elucidated, it is undoubtedly the predominant and most relevant organ manifestation determining morbidity and mortality. Therefore, early-onset ILD associated with systemic inflammation should always raise the suspicion of SAVI [10]. All previously reported SAVI patients with the heterozygous R281Q STING variant met the clinical or radiological criteria for ILD and suffered from advanced lung disease, with a lethal course in two cases. This underlines the unusual nature of the family reported here, with no signs of ILD in the mother of our index patient. Interestingly, an association of ILD and ANCA positivity, especially MPO ANCA, has been described in patients with vasculitis [11]. Notably, our index patient showed indeed high titers of p-ANCA and MPO ANCA, whereas his mother had only weakly positive p-ANCA levels. These findings are in line with the hypothesis of an anti-MPO driven autoimmune response as the cause of pulmonary tissue injury in ILD [12, 13]. However, whether anti-MPO has a direct pathogenetic in SAVI-associated lung disease, remains unknown. Given that SAVI cases with severe ILD but negative ANCA have been observed, additional factors are likely involved in ILD pathogenesis [4]. The ILD of our index patient also supports the observation in other SAVI patients, that the severity and progression of lung involvement seem to be independent of the genotype. Such a lack of genotype–phenotype correlation has also been observed for other disease-causing variants. For example, a 9-month-old infant carrying the R284S STING variant died of severe ILD [14], while the same disease-causing variant was found in a 25-year-old SAVI patient without any pulmonary involvement [4]. The respiratory impairment due to ILD in SAVI patients may be exacerbated by the increased susceptibility to infections commonly seen in SAVI, as was the case in our index patient with multiple hospitalizations because of pneumonia. Nonetheless, immunological investigations of our index patient revealed only mild abnormalities in leukocyte subpopulations without hypogammaglobulinemia which might reflect a subtle immunodeficiency. Increased susceptibility to respiratory infection might have been further aggravated by an infection-triggered bronchial hyperresponsiveness due to atopy.

In both patients, JAK inhibitor treatment was started to reduce uncontrolled type I IFN signaling which led to significant clinical improvement in the index patient. Unfortunately, interferon scores were only obtained after starting JAK inhibition in both the index patient and his mother. Interferon scores were repeatedly strongly elevated (97.06 and 155.01, normal range < 12.49) in the child, and negative or slightly increased in the mother. Notably, tofacitinib, given at a rather low dose, was less effective in the mother. For both ruxolitinib and tofacitinib, several case reports have described a good therapeutic response [15–19]. Interestingly, two case reports described inadequate symptom control with tofacitinib but symptom relief with ruxolitinib [20, 21], suggesting that this option could still be considered in the mother. Given the therapeutic benefit of JAK inhibition in patients with SAVI early diagnosis is of particular importance to prevent the development or progression of irreversible organ damage. In summary, our findings demonstrate that SAVI patients can present with unusual and rather unspecific manifestations and highlight the clinical significance of early and broad genetic testing in patients with rheumatologic manifestations.

## Data Availability

Not applicable.

## Abbreviations

ANA: Antinuclear antibodies
ANCA: Anti neutrophil cytoplasmic antibody
CBD: Cyclic dinucleotide-binding domain
cGAMP: 2’,3’-Cyclic Guanosine monophosphate-Adenosine monophosphate
cGAS: Cyclic GMP-AMP synthase
CT: Computed tomography
IFN: Interferon
ILD: Interstitial lung disease
IRF3: Interferon regulatory transcription factor 3
JAK: Janus kinase
MTX: Methotrexate
MPO: Myeloperoxidase
NSAID: Nonsteroidal anti-inflammatory drugs
PAH: Pulmonary arterial hypertension
PR3: Proteinase 3
RF: Rheumatoid factor
SAVI: STING-associated vasculopathy with onset in infancy
SLE: Systemic lupus erythematosus
STING: Stimulator of interferon genes
TBK1: TANK-binding kinase 1
TNF-α: Tumor necrosis factor-α
